# Seeds of *Zizyphus lotus*: *In Vivo* Healing Properties of the Vegetable Oil

**DOI:** 10.1155/2020/1724543

**Published:** 2020-06-08

**Authors:** C. Rais, C. Slimani, M. Benidir, L. Elhanafi, I. Zeouk, F. Errachidi, L. El Ghadraoui, S. Louahlia

**Affiliations:** ^1^Laboratory of Botany, National Agency of Medicinal and Aromatic Plants, P.O. Box 159, Taounate 34025, Morocco; ^2^Laboratory of Functional Ecology and Environment, Faculty of Sciences and Technology, Sidi Mohamed Ben Abdellah University, P.O. Box 2202, Route d'Imouzzer, Fez, Morocco; ^3^Laboratory of Engineering, Electrochemistry, Modeling and Environment, Faculty of Sciences Dhar Mahraz, Sidi Mohammed Ben Abdellah University, P.O. Box 1796, Fez-Atlas 30000, Morocco; ^4^Laboratory of Microbial Biotechnology, Faculty of Sciences and Technology, Sidi Mohamed Ben Abdellah University, P.O. Box 2202, Route d'Imouzzer, Fez, Morocco; ^5^Laboratory of Natural Resources and Environment, Polydisciplinary Faculty of Taza, USMBA, Route d'Oujda, B.P. 1223, 1223 Taza, Morocco

## Abstract

The present study has been undertaken in order to highlight the healing effect of *Zizyphus lotus* vegetable oil. The seeds of this plant contain an oil rate of 30%. The obtained results on the main elements composing the vegetable oil have shown that *Zizyphus lotus* vegetable oil has a low value of acidity index and it presents a not negligible degree of unsaturation. The value of the peroxide index of *Zizyphus lotus* vegetable oil is less than 10 which characterizes the most of conventional oils. Furthermore, the spectral analysis by gas chromatography has shown the presence of 53 majority and minority molecules. Thus, the evaluation of the healing activity of *Z. lotus* seed vegetable oil has demonstrated a highly significant effect against the negative control and silver sulfadiazine was used as conventional treatment for burns. Based on the obtained results, we can suggest that the oil extracted from the seeds of the studied plant could be used to cure wounds.

## 1. Introduction

From a climatic and ecological point of view, Morocco is one of the original countries of the Western Palearctic region. Hence, it is the second biologically diverse country in the Mediterranean basin after Turkey. Morocco is home to an important number of endemic species relating to the variety of natural areas. These species represent a vegetal heritage with great geographical or historical value; they are the originality of a vegetal landscape of which the aromatic and medicinal plants (AMP) occupy a large place. These are widely used in various fields including pharmacology and cosmetology; they play an important socioeconomic role.

The jujube (*Zizyphus lotus* L.), commonly called “Sedra” in Arabic and “Azouggart” in “Berber,” is an aromatic and medicinal plant widely used in herbal medicine by the local populations [1]. This plant species has numerous nutritiously, cosmetically, and medicinally interests. Its anti-inflammatory, analgesic, and antispasmodic activities have been demonstrated through numerous previous investigations including these of [2]. Its distribution area is extending all over the Mediterranean area.

During the last years, numerous phytochemical studies have been reported on *Zizyphus lotus* and they have shown the medical importance of this species. The effects against several diseases such as digestive disorders, liver problems, obesity, urinary disturbs, diabetes, skin infections, fever, diarrhea, and insomnia have been largely underlined [3–7]. Moreover, the fruits and leaves of *Z. lotus* are much known due to their composition in terms of flavonoids, sterols, tannins, saponins, and triterpenoids [8, 9]. Furthermore, *Z. lotus* fruits are appreciated by different animals such as sheep, cattle, camelids, and goat [10]. The current research studies about different pharmacological activities of this plant and its derivatives are very important for modern medicine. However, this species is often neglected or even forgotten. It is experiencing an increased degradation because of the negative impact of anthropogenic factors that are steadily growing (overgrazing and grubbing-up by farmers in order to change land use, etc.), and this plunder could jeopardize the sustainability of the species. Nevertheless, to our knowledge, no work was carried out on the healing effect of jujube.

Indeed, burns are traumatic pathologies responsible for significant morbidity and mortality. They are a public health problem, particularly with regard to their frequency, their potential gravity, the sequels that they can generate, and the high cost for their treatment and prevention. However, the use of numerous conventional products is often limited because of the varying levels of their effectiveness. The solution to these inconveniences is the use of natural products as an alternative based on traditional medicine. For a long time, these natural products have shown their efficiency to cure burns and scalds showing several interesting benefits in terms of availability, safety, and low cost price [11].

Due to the lack of information on pharmacological uses of *Z. lotus* seeds and in order to enhance the potentialities which peak this plant, we have reported the present work to highlight the effect of *Z. lotus* vegetable oil prepared from seeds on wound healing induced in mice.

## 2. Materials and Methods

### 2.1. Plant Material

The seeds of *Z. lotus* used in the present study have been collected from the region of Fez (Zouagha-Moulay Yaâcoub) in August 2016. Climatic data of the area of harvest are summarized in Table 1. The seeds have been isolated from fruit, ground in a mortar, and then sifted to obtain a very fine and homogeneous powder which has been extracted in order to obtain the vegetable oil and the extracts.

### 2.2. Extraction of Vegetable Oil from Seeds

10 g of the powder was macerated with 100 ml of hexane (ratio 10) under agitation at 500 rpm at room temperature for 24 hours. The resulting mixture was filtered using Whatman filter no. 1, and then the solvent was removed from the vegetable oil under vacuum using rotavapor. The obtained residue was stored in the dark in a refrigerator at 4°C until further use.

### 2.3. The Yield of Vegetable Oil

The yield was performed using the ratio of the vegetable oil weight after evaporation (**m**) to the weight of the dry vegetal matter (**ms**) used for extraction, multiplied by 100 [12]:

(1) $$\begin{array}{c} \mathrm{Rdt}\;\left(\%\right)=\left[\frac{m}{\mathrm{ms}}\right]\times \;100. \end{array}$$

### 2.4. Chemical Composition of the Vegetable Oil

The study of the main elements composing the vegetable oil is very interesting to understand this essence's wound healing. Three major elements have been identified: iodine index, acidity index, and peroxide index. These elements could give an idea about the composition of vegetable oil which facilitates the comprehension of possible pharmacological effects including wound healing.

#### 2.4.1. Iodine Index

Iodine index is defined as the number of grams of iodine fixed by 100 grams of vegetable oil. Thus, 0.2 g of fat was solubilized in 10 ml of chloroform [13, 14]. After homogenizing the solution, 2 ml was introduced into a dry Erlenmeyer flask to which it was added 2 ml of WIJS reagent. The control was prepared using a mixture of chloroform and WIJS (2 : 2). Both solutions (real and control) were mixed, placed in the dark for 60 min, and agitated from time to time. Then, 2 ml of KI solution was added (10%) and agitated for 2 min. The excess iodine I_2_ was determined using 0.2 M sodium thiosulfate that discolors the content. Volumes V′i (control) and Vi (real) of thiosulfate corresponding to each titration (test) have been also noticed.

Iodine index being related to the unsaturated nature of fat allows calculating the number of double bonds per fatty acid molecule (equation (2)):

(2) $$\begin{array}{c} \mathrm{Ii}=N\;\times \;\left(V^{\prime }i\;\mathrm{test}\;–\;\mathrm{Vi}\;\mathrm{real}\right)\;\times \frac{127}{P\;\times \;10}, \end{array}$$

where **V′** is the volume of sodium thiosulfate of the used normalized solution; **N** is the normality of sodium thiosulfate; **P** is the weight in g of the test sample of the studied fat.

The number of double bonds was determined using the following formula:

(3) $$\begin{array}{c} n=\frac{\mathrm{Ii}\;\times \;M}{254\;\times 100}, \end{array}$$

where **n** is the number of double bonds of fatty acid and **M** is the molecular mass of fatty acid.

#### 2.4.2. Acidity Index

The determination of this index was performed through dissolution of 0.25 g of vegetable oil in 10 ml of ethanol in which 5 ml of alcoholic KOH was added (ethanol at 10%) [13, 14]. After homogenization, 2 drops of phenolphthalein were added. The titration was carried out with 0.2 N sulfuric acid until the pink coloration of persistent phenolphthalein for at least 10 seconds [13, 14]. The control was prepared using all the solutions of the real test except the oil.

The acidity index was calculated as follows:

(4) $$\begin{array}{c} \mathrm{IA}\;=\frac{N\;\times \;M\;\times \;\left(V^{\prime }\mathrm{control}\;-\;V^{\prime }\mathrm{real}\right)}{P}, \end{array}$$

where **V′** is the volume of the used sulfuric acid, **N** is the normality of the solution, and **P** is the mass of the test sample.

#### 2.4.3. Peroxide Index

The peroxide index is defined as the number of milliequivalents of active oxygen per kilogram of fatty acid. This index allows the evaluation of oxidation degree of the unsaturated fatty acids of the fat. Indeed, 10 ml of chloroform was added to 1 g of the sample [13, 14]. The test portion dissolves rapidly with stirring. 15 ml of acetic acid was added and then 1 ml of potassium iodide saturated solution was added. After agitation for 1 min, the sample was being left sitting for 5 min in the dark, and then 75 ml of distilled water was added. The obtained mixture was dosed using sodium thiosulfate solution (0.005 N) and starch. The color change (from blue to colorless) indicated the end of dosage. The control was prepared in the same operational conditions containing all the solutions of the real test except the vegetable oil.

Peroxide index (**Ip**) is expressed as milliequivalents of active oxygen/kg and obtained by the following equation:

(5) $$\begin{array}{c} \mathrm{Ip}=\frac{\left(V^{\prime }\mathrm{real}–V^{\prime }\mathrm{control}\right)\times T\times 1000}{M}, \end{array}$$

where **V′** is the volume (ml) of sodium thiosulfate normalized solution used, **T** is the degree of normality of used thiosulfate solution, and **M** is the mass (g) of the test sample.

### 2.5. Gas Chromatography

The fatty acid methyl ester (FAME) profile was determined after basic transesterification. The reaction was catalyzed by 2% KOH (w : w) in methanol 1 : 20 for 1 hour under ultrasonic (Branson Sonifier 450) (40 kHz) at 40°C. FAME profile was characterized by gas chromatography (GC) (Agilent 7890A Series GC) coupled to mass spectrometry (MS) equipped with multimode injector and 5 MS column with a dimension of 30 m × 250 *μ*m × 0.25 *μ*m and electron impact ionization according to our protocol.

Two microliters of FAME solubilized in chloroform was injected into the column by splitless mode using helium as carrier gas at 1.5 ml/min. The ion source and quadruple temperatures were 230°C and 150°C, respectively. The oven temperature program was started at 70°C and maintained 1 min, increased at 20°C/min until 120°C, then held one minute before to be increased until 200°C by 30°C/min and held one minute then, increased at 250°C at 10°C/min and held one minute, then increased until 305°C at 5°C/min, and finally kept constant for 3 min. FAME composition was calculated as percentage of the total FAMEs present in the sample, determined from the peak areas. Detection was done using full scan mode between 35 and 600 *m*/*z* and with gain factor 5, and the identification was performed using NIST 2014 MS Library.

### 2.6. *In Vivo* Test of the Vegetable Oil on Thermal Burns of Mice

In order to highlight the effect of *Z. lotus* vegetable oil on thermal burns of mice, we have realized burns on the dorsal part of the animal, and then we have applied the treatment using the vegetable oil of *Z. lotus* seeds. The observed effect was compared to silver sulfadiazine 1% as standard treatment on burn care units. To achieve this, we have used 18 mice which were from the pet store of pharmacological service in the Faculty of Sciences and Techniques-Fez. Mice have been placed individually in polystyrene cages with free access to water and food.

Tested animals were divided into 3 lots each comprising 6 mice:

  Lot negative control (NC): without treatment
  Lot positive control (PC): treatment by silver sulfadiazine 1%
  Lot seeds' oil (SO): treatment using vegetable oil of *Z. lotus* seeds

### 2.7. Mice Burn Model

**Figure 1:**
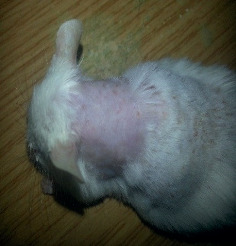
Shaving of the target site.

**Figure 2:**
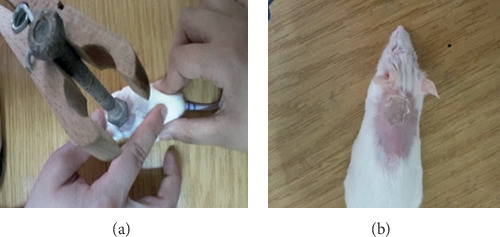
(a) Burn performance; (b) aspect of burn immediately after induction.

After general anesthesia using ether, we have shaved the dorsal part of the animal in which we have induced thermal burns. The choice of this body's part could be justified by the facility of access (Figure 1). Burns have been applied using a counterweight in a round contact surface with 1 cm in diameter (Figure 2). This one was heated at 100°C and then applied for 20 seconds in the prepared part. This technique was performed as described by Cai et al. [15].

### 2.8. Treatment Application

**Figure 3:**
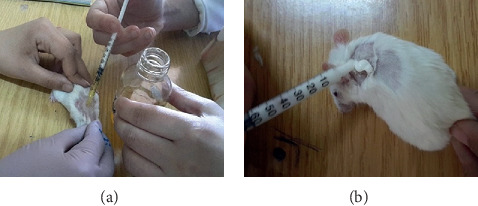
Treatment application: (a) seeds' oil and (b) cream (sulfadiazine). Note: photographs have been taken from the same angle in order to minimize the camera angle variations which could cause an underestimation of the wound surface.

After burns induction, treated animals have received treatments (Figure 3):

  Lot PC: using 0.1 ml of silver sulfadiazine spread out on the wound
  Lot SO: using 0.1 ml of *Z. lotus* vegetable oil.

Treatments were applied one time per day, since the experiment beginning until the cure of treated animals. Mice of negative test lot do not receive any treatment, and they have suffered the same degree of stress associated with the application of treatment.

The control of wound evolution was undertaken one time per day by taking photos. The documentation of wound healing evolution through photography has the advantage of avoiding contact with the wound, thus a permanent registration not just of the wound size but also its appearance. The experimental procedure was performed as described by Chang et al. [16].

### 2.9. Ethical Note

This study was performed under the proper legislation of the Moroccan law and was approved by the Ethical Committee of Moroccan Association for Animal's Health and Laboratory of Functional Ecology and Environment, Biology Department, Faculty of Science and Technology-Fez. Also, during the study, all tests and analyses were recorded with minimum disturbance in conformity with Moroccan legislation. Finally, animal's health was diagnosed by animal rearing cell in Biology Department.

### 2.10. Healing Evolution

The study of burns healing evolution was performed using digital planimetry. The general principle of this technique is taking pictures of wounds at regular intervals and then following the evolution of their surface. The focus of the pictures and resolution were adjusted using the software “ImageJ.” This method is rapid, precise, and objective.

#### 2.10.1. Calculation of Contraction Percentage

In order to determine the percentage of wound contraction, the techniques reported by Gopinath et al. [17] have been used; it consists in calculation of surface means of wounds and its comparison with the initial burn using the following equation:

(6) $$\begin{array}{c} \%\;\mathrm{de}\;\mathrm{contraction}=\frac{\mathrm{Tpi}\;\mathrm{J0}–\mathrm{Tp}\;\mathrm{to}\;\mathrm{Jn}}{\mathrm{Tpi}}\times 100, \end{array}$$

where **Tp** is the size of the wound and **Tpi** is the size of the initial wound.

### 2.11. Calculation of Reepithelialization Period

The period of reepithelialization was determined by the number of days required for the fall of the eschar and total closure of the wound that leaves behind, which did not leave residual injury [18, 19].

### 2.12. Statistical Analysis

Data were subjected to two-way analysis of variance (ANOVA) in order to determine significant differences among the treatments. The data were processed using the “SYS-TAT 12” software. A mean comparison test was performed whenever there was a significant factor effect studied by ANOVA.

## 3. Results and Discussion

### 3.1. Yield of Vegetable Oil Prepared from Seeds

*Zizyphus lotus* seeds contain an oil rate of 30%, and this result is in agreement with the investigation reported by Hachimi et al. [20], in which they have demonstrated that populations of jujube contain an excellent extraction oil rate of 29.25%, followed by pomegranate (23.39%). The most weak value is observed for the varieties of Barbary fig with 8.74%.

### 3.2. Chemical Characterization of Seeds' Oil

#### 3.2.1. Iodine Index

The determination of the iodine index allowed us to highlight the degree of unsaturation of the oil. Thus, the obtained value in our study is of the order of 69.85 g of iodine/100 g of fat body comparing it to that of olive oil which varies between 70 and 94 g of iodine/100 g of oil [21]. This allows us to demonstrate that *Z. lotus* vegetable oil presents an unsaturation degree not neglected. This finding is in accordance with the previous work of some authors [20] having noticed that the vegetable oil of *Z. lotus* is unsaturated with the presence of four majority fatty acids, namely, oleic acid (62.49%), linoleic acid (16.31%), palmitic acid (10.27%), and stearic acid (6.48%). Overall, in these vegetable oils, unsaturated fatty acids are present in important quantity (81.83%) in comparison with saturated fatty acids. The determination of the iodine index allowed us to notice that the number of double bonds of this vegetable oil is 1 for 0.04 gram of oil.

#### 3.2.2. Acidity Index

The quality of vegetable oil is determined by the value of acidity index. This parameter characterizes the purity and the stability of oils at ambient temperature. The obtained results have demonstrated that *Z. lotus* oil presents a weak acidity index of 0.08 mg KOH/g fatty body. It is inferior to the most of usual oils, such as soybean oil (max 3 mg/g) and olive oil that varies between 0.37 and 0.49 mg/g of oil [21]. The low value of acidity index confers a good stability to oil.

#### 3.2.3. Peroxide Index

The peroxide index depends on conservation conditions and extraction modes. It seems that this index has a significant sensibility and represents a very useful criterion for assessing the first stages of oxidative deterioration. The obtained value of the peroxide index in the current survey is 8 meq·O_2_/kg of oil (Table 2). This value is superior to that of olive oil (1.14 meq·O_2_/kg) [21]. However, the value obtained in our study is inferior to 10 meq·O_2_/kg which characterizes the most of the conventional oils [22].

### 3.3. Gas Chromatography

**Figure 4:**
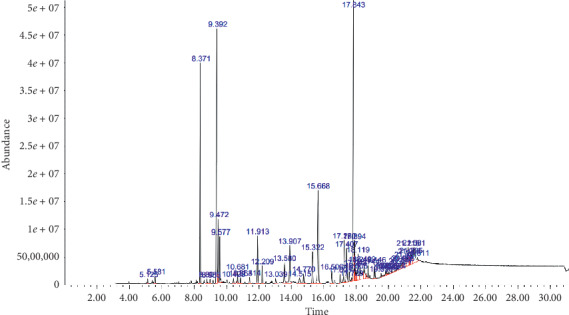
Burns of mice treated using vegetable oil of *Z. lotus* seeds.

The spectral analysis by gas chromatography has shown the presence of 53 majority and minority molecules (Figure 4). The majority compounds are, namely, linoleic acid with 37%, palmitic acid with 28%, and oleic acid with 25%. Consequently, minority compounds represent 10%.

In order to highlight the effect of *Z. lotus* vegetable oil on thermal burns of mice, numerous parameters have been studied. The observed effect is compared with that obtained by silver sulfadiazine 1% as standard treatment on burn care units.

### 3.4. Average Reepithelialization Times

The average complete reepithelialization times of the burns of the three lots of studied mice (seeds' oil, positive control, and negative control) are presented in Table 3. The obtained results have shown that the seeds' oil (SO) of *Z. lotus* has a beneficial effect on healing of lesions caused by burns in the treated mice. Moreover, the average duration of total healing of the burns of treated mice with SO is 15.67 ± 9.24 days. This duration is very inferior to the average complete reepithelialization duration of positive (PC) and negative control (NC) of mice. The statistical treatment of the obtained results has revealed highly significant differences between different groups of studied mice (SO, PC, and NC) (ANOVA: *F* = 120.77; ddl = 2; *P* < 0.001).

### 3.5. Variation of the Average Surface of Burns and the Percentage of Contraction

**Figure 5:**
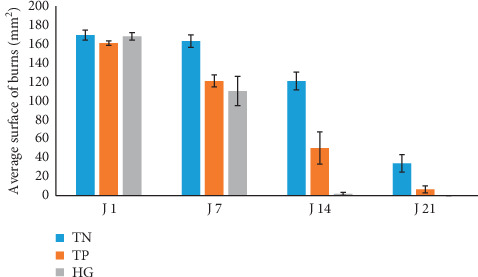
Evolution of the average surface of burns caused in the three lots (SO, NC, and PC). Vertical bars correspond to the SE (*n* = 6).

**Figure 6:**
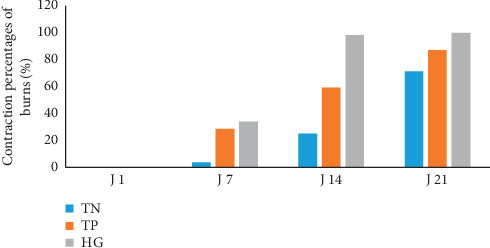
Contraction percentages of burns in the three lots of treated mice (SO, NC, and PC). Vertical bars correspond to the SE (*n* = 6).

**Figure 7:**
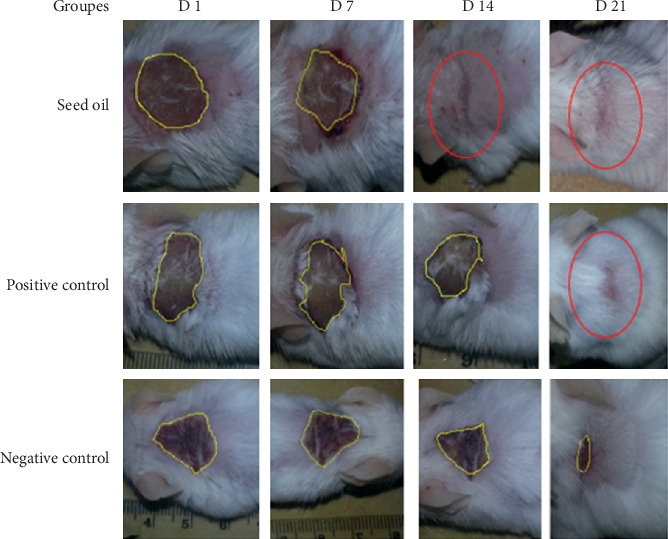
Aspects of burns in the three lots of treated mice (SO, NC, and PC) from 1^st^ to 21^st^ day.

These parameters are presented in Figures 5 and 6. According to the obtained findings, we have noticed that the surface of burns decreases following applied treatments. This decrease varies upon the nature of the reported treatment. During the first days (from D1 to D7) of post-burn treatment which comprises the inflammatory phase of healing, burns of SO lots have registered decreases in their highest surfaces (from 168 ± 4.05 mm^2^ to 110 ± 15.41 mm^2^) with a contraction percentage of 34.01%. However, the reductions in the surfaces of the lowest burns have been noticed in lot of NC, from 169.3 ± 5.37 to 163 ± 6.57 mm^2^ with a low contraction percentage of 3.78%. From the 14^th^ day of treatment, the difference in size between the burns of the three lots of studied mice was accentuated. All the lesions caused in the mice of lot SO were totally healed. The NC group presents the most important average surface of 121 ± 9.30 mm^2^ with a contraction percentage of 25.11%. Thus, the first cases of total healing of burns were observed up to 28^th^ day (Figure 7). The analysis of the relative variance of burns surfaces has demonstrated a very high significant effect between the three studied lots (ANOVA: ddl = 2; *F* = 39.35; *P* < 0.001).

## 4. Discussion

The obtained findings in the current study are in agreement with several other works that have shown the therapeutic virtues of *Zizyphus lotus* as one of the most interesting aromatic and medicinal plants [23, 24]. Moreover, *Z. lotus* oil has displayed a promising anti-inflammatory activity [25]. These results confirm the traditional uses of *Zizyphus lotus*. It is known that silver sulfadiazine (SS) is used for its antimicrobial effect in order to fight a possible infection whose prevention is necessary for good healing of wounds [26]. Another advantage is its simple use and efficacy in the treatment of skin ulcers [27]. Despite these many advantages, numerous negative side effects of this medical drug have been reported including renal toxicity and leucopenia which limit its use for long periods of treatment and over extended wounds [28], and delayed healing cases have been also reported [29]. In addition, allergic reactions to silver sulfadiazine limit its use for some patients [30]. The healing effect of *Z. lotus* oil was more marked during the inflammatory phase of wound healing, where wounds of the treated lot showed very large reductions in size compared with those of PC and NC, and this could be explained by its anti-inflammatory activity.

These results are in disagreement with those found by Abdeldjelil [31], and he has shown that lentisque oil has a stronger effect during the proliferative phase of healing. As any natural product, the healing effect of this vegetable oil seems to be due to the various chemical constituents involved in its composition. According to several authors, natural healing products manifest their effects through one of the following mechanisms: antimicrobial effect, anti-inflammatory effect, antioxidant, stimulation of collagen synthesis, and cell proliferation [32]. In the case of *Zizyphus lotus* oil, its richness in bioactive molecules such as fatty acids, triglycerides, phytosterols, tocopherols, and polyphenols could explain the observed healing effect in the current survey [20, 33]. Under the effect of burns, the structure of the upper layers of the epidermis is damaged and the skin loses its protective hydrolipidic film. As a result, the skin performs less of its role as a barrier and becomes on the one hand a gateway for bacteria, and on the other hand, it retains less water than it contains [34]. The protective effect of *Z. lotus* vegetable oil on burned skin is therefore directly related to its high lipid content. This finding has been revealed previously [20] and showed that the vegetable oil prepared from *Z. lotus* seeds is very rich in unsaturated fatty acids (UFAs) with 81.83% in comparison with saturated fatty acids (SFAs). These lipids would limit the transcutaneous loss of water. The addition of antioxidants to the list of treatments of patients with burns has beneficial effects such as reducing the incidence of wound infection and reducing healing time compared to other conventionally treated patients without the addition of antioxidants [35]. Based on the results reported by Chouaibi et al. [33], the oil of *Z. lotus* oil contains tocopherols, polyphenols (144.83 mg/kg of oil), and carotenoids (6.34 mg/kg of oil). These compounds have a hydroxyl function (−OH) that allows them to trap free radicals, thus giving them an important antioxidant power. This could explain the total healing effect of lots treated by *Z. lotus* during a period less than that of NC and PC.

## 5. Conclusion

The analysis of obtained data in the current study is encouraging and shows that the use of *Zizyphus lotus* as an anti-infectious agent in traditional environments is justified and that it should be studied more widely in order to explore its potential in the treatment of infectious diseases. Furthermore, the obtained results allow us to conclude that the oil extracted from seeds of *Zizyphus lotus* could be used to cure burns.

## Figures and Tables

**Table 1:** Principal climatic data of the studied station.

Climatic data	Station
	FES
Latitude	34°02′13 N
Longitude	4°59′59 W
Altitude	403
Rainfall (mm/year)	375
Bioclimatic stage	Semiarid

Note: N: north; W: west.

**Table 2:** Chemical parameters of *Z. lotus* vegetable oil.

Index	Maceration/hexane
	values
Acidity index	0.08 (mg·KOH/g·mg)
Iodine index	69.85 (g/100 g·mg)
Peroxide index	5 (meq·O_2_/kg)

**Table 3:** Average duration (days) of complete reepithelialization of burns of mice in different lots (SO, PC, and NC).

Lots		
NC	SO	PC
28.33 ± 7.13 (D)	15.67 ± 9.24 (D)	22.67 ± 8.58 (D)

## Data Availability

No data were used to support this study.
